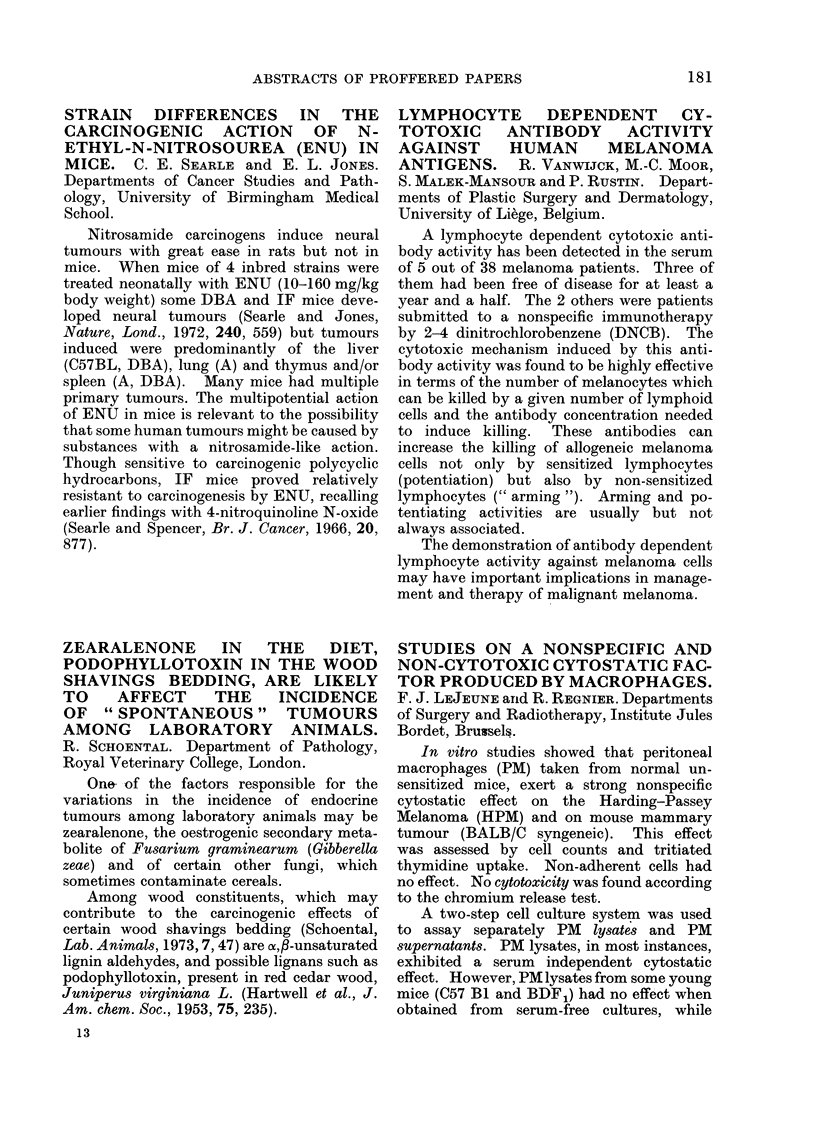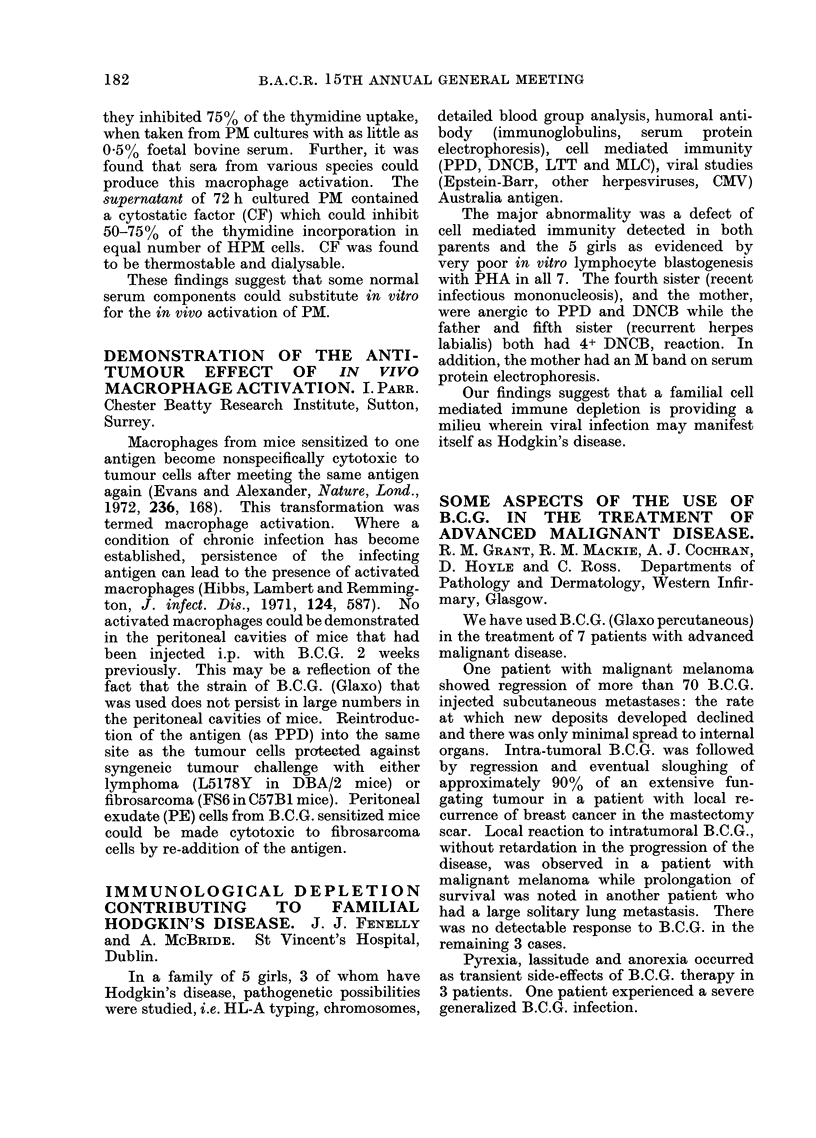# Proceedings: Studies on a nonspecific and non-cytotoxic cytostatic factor produced by macrophages.

**DOI:** 10.1038/bjc.1974.161

**Published:** 1974-08

**Authors:** F. J. LeJeune, R. Reginier


### STUDIES ON A NONSPECIFIC AND NON-CYTOTOXIC CYTOSTATIC FACTOR PRODUCED BY MACROPHAGES.

F. J. LeJeune and R. Regnier. Departments of Surgery and Radiotherapy, Institute Jules Bordet, Brussels.

*In vitro* studies showed that peritoneal macrophages (PM) taken from normal unsensitized mice, exert a strong nonspecific cytostatic effect on the Harding–Passey Melanoma (HPM) and on mouse mammary tumour (BALB/C syngeneic). This effect was assessed by cell counts and tritiated thymidine uptake. Non-adherent cells had no effect. No *cytotoxicity* was found according to the chromium release test.

A two-step cell culture system was used to assay separately PM *lysates* and PM *supernatants*. PM lysates, in most instances, exhibited a serum independent cytostatic effect. However, PM lysates from some young mice (C57 Bl and $BDF_1$) had no effect when obtained from serum-free cultures, while they inhibited 75% of the thymidine uptake, when taken from PM cultures with as little as 0·5% foetal bovine serum. Further, it was found that sera from various species could produce this macrophage activation. The *supernatant* of 72 h cultured PM contained a cytostatic factor (CF) which could inhibit 50–75% of the thymidine incorporation in equal number of HPM cells. CF was found to be thermostable and dialysable.

These findings suggest that some normal serum components could substitute *in vitro* for the *in vivo* activation of PM.